# Partial restoration of protein synthesis rates by the small molecule ISRIB prevents neurodegeneration without pancreatic toxicity

**DOI:** 10.1038/cddis.2015.49

**Published:** 2015-03-05

**Authors:** M Halliday, H Radford, Y Sekine, J Moreno, N Verity, J le Quesne, C A Ortori, D A Barrett, C Fromont, P M Fischer, H P Harding, D Ron, G R Mallucci

**Affiliations:** 1 Medical Research Council Toxicology Unit, Hodgkin Building, University of Leicester, Leicester, UK; 2 Cambridge Institute for Medical Research, University of Cambridge, Cambridge Biomedical Campus, Cambridge, UK; 3 Department of Histopathology, University Hospitals of Leicester NHS Trust, Leicester, UK; 4 Centre for Analytical Bioscience, School of Pharmacy, University of Nottingham, Nottingham, UK; 5 Division of Medicinal Chemistry & Structural Biology, School of Pharmacy, Centre for Biomolecular Sciences, University of Nottingham, Nottingham, UK; 6 The Wellcome Trust MRC Institute of Metabolic Science and NIHR Cambridge Biomedical Research Centre, Cambridge, UK; 7 Department of Clinical Neurosciences, Cambridge Biomedical Campus, University of Cambridge, Cambridge, UK

## Abstract

Activation of the PERK branch of the unfolded protein response (UPR) in response to protein misfolding within the endoplasmic reticulum (ER) results in the transient repression of protein synthesis, mediated by the phosphorylation of the alpha subunit of eukaryotic initiation factor 2 (eIF2*α*). This is part of a wider integrated physiological response to maintain proteostasis in the face of ER stress, the dysregulation of which is increasingly associated with a wide range of diseases, particularly neurodegenerative disorders. In prion-diseased mice, persistently high levels of eIF2*α* cause sustained translational repression leading to catastrophic reduction of critical proteins, resulting in synaptic failure and neuronal loss. We previously showed that restoration of global protein synthesis using the PERK inhibitor GSK2606414 was profoundly neuroprotective, preventing clinical disease in prion-infected mice. However, this occured at the cost of toxicity to secretory tissue, where UPR activation is essential to healthy functioning. Here we show that pharmacological modulation of eIF2*α*-P-mediated translational inhibition can be achieved to produce neuroprotection without pancreatic toxicity. We found that treatment with the small molecule ISRIB, which restores translation downstream of eIF2*α*, conferred neuroprotection in prion-diseased mice without adverse effects on the pancreas. Critically, ISRIB treatment resulted in only partial restoration of global translation rates, as compared with the complete restoration of protein synthesis seen with GSK2606414. ISRIB likely provides sufficient rates of protein synthesis for neuronal survival, while allowing some residual protective UPR function in secretory tissue. Thus, fine-tuning the extent of UPR inhibition and subsequent translational de-repression uncouples neuroprotective effects from pancreatic toxicity. The data support the pursuit of this approach to develop new treatments for a range of neurodegenerative disorders that are currently incurable.

The protein misfolding neurodegenerative disorders, which include Alzheimer's (AD) and Parkinson's (PD) diseases, amyotrophic lateral sclerosis (ALS), as well as the rare prion disorders, have in common the presence of aggregates of misfolded proteins in the brain associated with neuronal loss. Individual diseases are characterised by the accumulation of disease-specific proteins and stereotyped clinicopathological phenotypes. While specific toxic effects for individual proteins, including oligomeric and aggregated forms, have been reported (see Winklhofer *et al.*^1^ for review), the mechanisms of neuronal death remain unclear and as a result effective therapies have been elusive. However, it is likely that common mechanisms as well as disease-specific ones lead to neurodegeneration in these disorders. Disruption of cellular processes such as protein recycling and autophagy^2^ and mitochondrial dysfunction^3^ contributes to the pathogenesis of several diseases. Most recently, the concept of disruption of proteostasis through endoplasmic reticulum (ER) stress and the attendant unfolded protein response (UPR) has emerged as a major contributor to neurodegenerative diseases.^4, 5^

Under physiological conditions, UPR activation ensures proteostasis is maintained through a combination of translational and transcriptional responses triggered by the accumulation of misfolded proteins in the ER.^6^ One branch of the UPR results in the transient repression of protein synthesis via the phosphorylation of pancreatic ER kinase (PERK), which in turn phosphorylates the alpha subunit of eukaryotic initiation factor 2 (eIF2). eIF2*α*-P prevents the formation of ternary complex, blocking translation at the level of initiation. Dephosphorylation of eIF2*α*-P by its specific phosphatase GADD34 allows protein synthesis to resume.^7^

We previously showed that, in prion-diseased mice, rising levels of misfolded prion protein (PrP) promote sustained elevation of eIF2*α*-P in neurons, leading to the protracted, uncompensated decline in global translation rates, resulting in the loss of crucial proteins that contributes to synaptic failure and neuronal death. Lowering eIF2*α*-P levels by genetic manipulations restored translation and was profoundly neuroprotective, prolonging survival in prion-infected mice,^8^ leading to the prediction that the pharmacological inhibition of PERK kinase activity to reduce eIF2*α*-P would be equally effective in preventing disease. Correspondingly, the small molecule GSK2606414,^9^ a highly specific PERK inhibitor, completely restored vital protein synthesis rates, abrogating neurodegeneration and preventing clinical disease in prion-infected mice.^8, 10^ Critically, these effects were downstream of prion replication and PrP accumulation, and therefore not specific to prion disease. Activated (phosphorylated) PERK-P and eIF2*α*-P are found in brains of patients with AD, PD, prion diseases and related disorders,^11, 12, 13, 14, 15^ and in several mouse models of different neurodegenerative diseases.^16, 17, 18, 19^ Hence, the development of a small molecule targeting the UPR as a generic treatment for the prevention neurodegeneration, independently of disease-specific misfolded protein, is of intense interest. Unfortunately, however, despite excellent neuroprotection in the brain, treatment with GSK2606414 in prion-diseased mice was associated with toxicity leading to weight loss and mild hyperglycemia,^10^ a predicted consequence of PERK inhibition in the pancreas.^20^ The pancreas has an extensive secretory protein synthesis load, and therefore requires some degree of eIF2*α*-P-mediated translational repression to survive ER stress. These findings beg the question of whether restoration of protein synthesis to the level required to prevent neurodegeneration is obligatorily linked to dose-limiting toxicity, which is central to the further pursuit of this approach to therapy.

eIF2*α* phosphorylation is a hub for signaling through other eIF2*α* kinases, as part of the broader integrated stress response (ISR). This leads to the inhibition of protein synthesis and increased expression of the transcription factor ATF4 and associated downstream signaling events, in response to other cellular stresses mediated by various eIF2*α* kinases, including PERK^6^ (Figure 1). Further, the regulation of protein synthesis rates through eIF2*α*-P has a key role in learning and memory.^21^ Recently, the small molecule *N*,*N*'-*trans*-(cyclohexane-1,4-diyl)-*bis-*(2-(4-chlorophenoxy)acetamide (ISRIB), which prevents ISR-mediated translational inhibition downstream of eIF2*α*-P (Figure 1), was shown to improve memory in wild-type mice.^22^ Importantly, these beneficial effects of ISRIB accrued without reported toxicity. We therefore asked if the use of ISRIB, as an alternative approach to PERK inhibition with GSK2606414, was neuroprotective in prion-diseased mice and without adverse effects. We tested the effect of treatment with ISRIB on clinical disease and neuropathology in prion-infected animals, and examined the pancreas for comparison with the effects of treatment with GSK2606414. We measured protein synthesis rates to compare the effects of the two compounds on UPR/ISR-mediated translational attenuation to determine whether this can be safely manipulated for the prevention of neurodegeneration without problematic toxicity.

## Results

### ISRIB penetrates the blood–brain barrier

We synthesized ISRIB as described,^22^ and confirmed penetration of the blood–brain barrier and measured mean brain:plasma ratios at various doses in wild-type mice. Intra-peritoneal administration of 0.25 mg/kg of *trans*-ISRIB (henceforth termed simply ISRIB), as used by Sidrauski *et al.*^22^ to enhance memory in mice, gave a favorable pharmacokinetic profile (Table 1), so this dose was used for treatment.

### ISRIB reduces ATF4 levels and partially restores protein synthesis rates in brains of prion-diseased mice

We used tg37^+/−^ mice^23^ infected with Rocky Mountain Laboratory (RML) prions, as used in previous studies.^8, 10, 23, 24, 25, 26^ These mice show PERK/eIF2*α*-mediated translational repression at 9 weeks post inoculation (w.p.i.) and develop neuronal loss progressing to clinical signs and death at ~12 w.p.i.^8^ Prion-infected mice were treated daily with 0.25 mg/kg of ISRIB or vehicle alone from 7 w.p.i., when synapse loss is established as previously performed with GSK2606414.^10^ Uninfected animals were included as controls. Mice were analyzed biochemically at 9 w.p.i., when prion-mediated UPR activation occurs. High levels of eIF2*α*-P were seen by western blotting of hippocampal protein samples from both ISRIB and vehicle-treated animals, but ISRIB treatment reduced levels of ATF4 markedly, consistent with its point of action downstream of eIF2*α*-P, as previously described^22^ (Figure 2a). ISRIB treatment reversed UPR/ISR-mediated translational repression and significantly increased global protein synthesis rates in prion-diseased mice to ~70% of levels seen in uninfected controls, compared with rates of only 40% in vehicle-treated animals, as measured by incorporation of [^35^S]-methionine into proteins in hippocampal slices (Figure 2b).

### ISRIB is neuroprotective in prion-diseased mice

Clinical prion disease in mice is diagnosed by a combination of early indicator and later confirmatory signs, the latter of which are usually correlated with advanced neuronal loss.^10^ The clinical course and incubation period are consistent for specific strains of prions and mice. We found that none (0/12) of the ISRIB-treated animals, but all (9/9) vehicle-treated mice developed confirmatory signs of prion disease by 12 w.p.i. on clinical observation (Table 2). Consistent with this, neuropathological examination confirmed that ISRIB treatment prevented neuronal loss in the hippocampus and reduced typical prion spongiform pathology, in marked contrast to extensive neurodegeneration and spongiform degeneration due to untreated prion disease in vehicle-treated mice (Figure 3a). Levels of total PrP and protease resistant PrP (PrP^Sc^) were similar in both groups of mice (Figure 3b), as ISRIB acts downstream of prion replication. Notably, ISRIB significantly increased survival in prion-infected mice from 84±3 days post infection in vehicle-treated mice (*n*=9) to 96±4 days (*n*=12) (Figure 4) at which point, however, despite being in good health, the animals had to be killed because of loss of 20% of body weight, as per protocol. At this stage, no signs of prion disease either clinically or in terms of neurodegeneration were found.

### ISRIB is not toxic to the pancreas, in contrast to the PERK inhibitor GSK2606414

Our previous results using the PERK kinase inhibitor, GSK2606414, similarly led to weight loss after chronic treatment in prion-diseased mice.^10^ PERK^−/−^-knockout mice show early postnatal lethality and exocrine pancreatic insufficiency,^20^ and it is predictable that the pancreas is vulnerable to PERK inhibition. Owing to its massive ER-protein synthesis load, pancreatic tissue needs a degree of eIF2*α*-P-mediated translational repression to survive ER stress, hence it might be expected that ISRIB would be similarly toxic to the pancreas. Pancreatic exocrine damage would be predicted to lead to weight loss and inanition, and endocrine damage to glucose intolerance and diabetes.

To understand the systemic effects of both compounds in causing weight loss, we treated another group of prion-infected mice for 5 weeks with either GSK2606414 or ISRIB and examined the pancreas and measured blood glucose levels. The latter were unaffected by ISRIB treatment but were mildly but significantly elevated by GSK2606414 (Figure 5a), consistent with previous findings.^10^ However, morphological examination of the pancreas showed that chronic GSK2606414 treatment was toxic, causing ~50% reduction in pancreatic weight (Figure 5b) and extensive destruction of exocrine acinar pancreatic tissue (Figure 5c). Critically, ISRIB treatment produced no detectable pancreatic toxicity; pancreatic weights were normal (Figure 5b) and structure and integrity of exocrine and endocrine pancreatic tissues was preserved (Figure 5c). Thus, while reversal of translational inhibition by both GSK2606414 and ISRIB is neuroprotective, the two compounds have very different adverse effect profiles on the pancreas.

### ISRIB is a partial inhibitor of the integrated stress response

**Figure 1:**
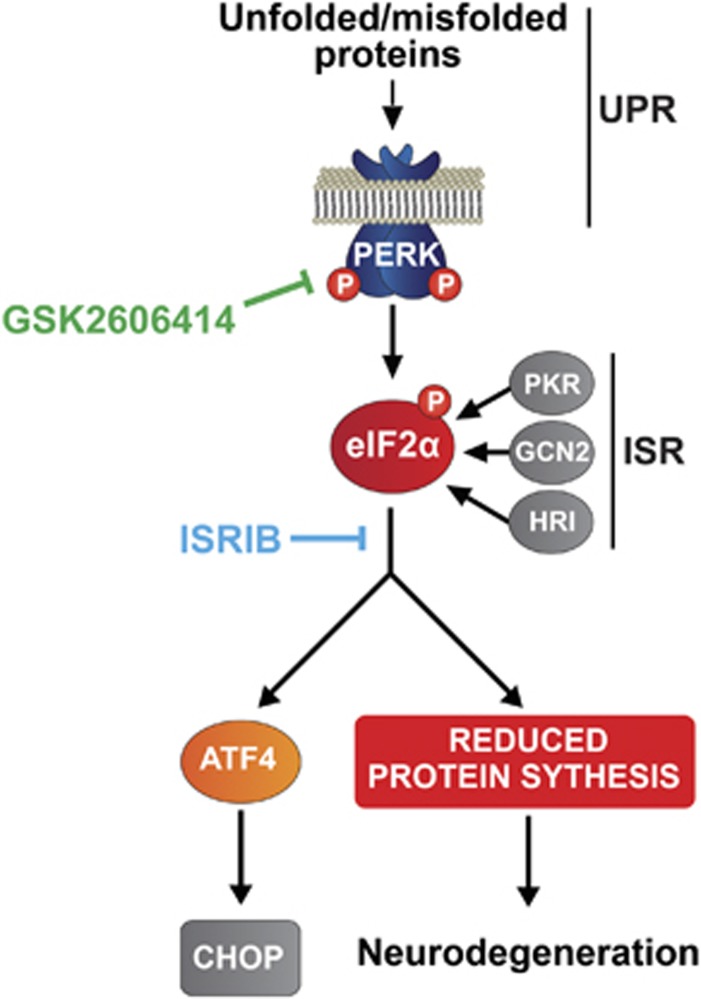
Scheme of the PERK branch of the UPR. Unfolded proteins activate PERK, which phosphorylates eIF2*α*. This represses translation at the level of initiation. Some proteins, however, escape this repression and are preferentially translated after eIF2*α* phosphorylation, such as ATF4, which leads to the translation of the pro-apoptotic CHOP. Chronic translational repression leads to neurodegeneration in prion disease. Signaling through the integrated stress response (ISR) can also lead to eIF2*α*-P and translational repression. The sites of action of GSK2606414 and ISRIB are shown

**Figure 2:**
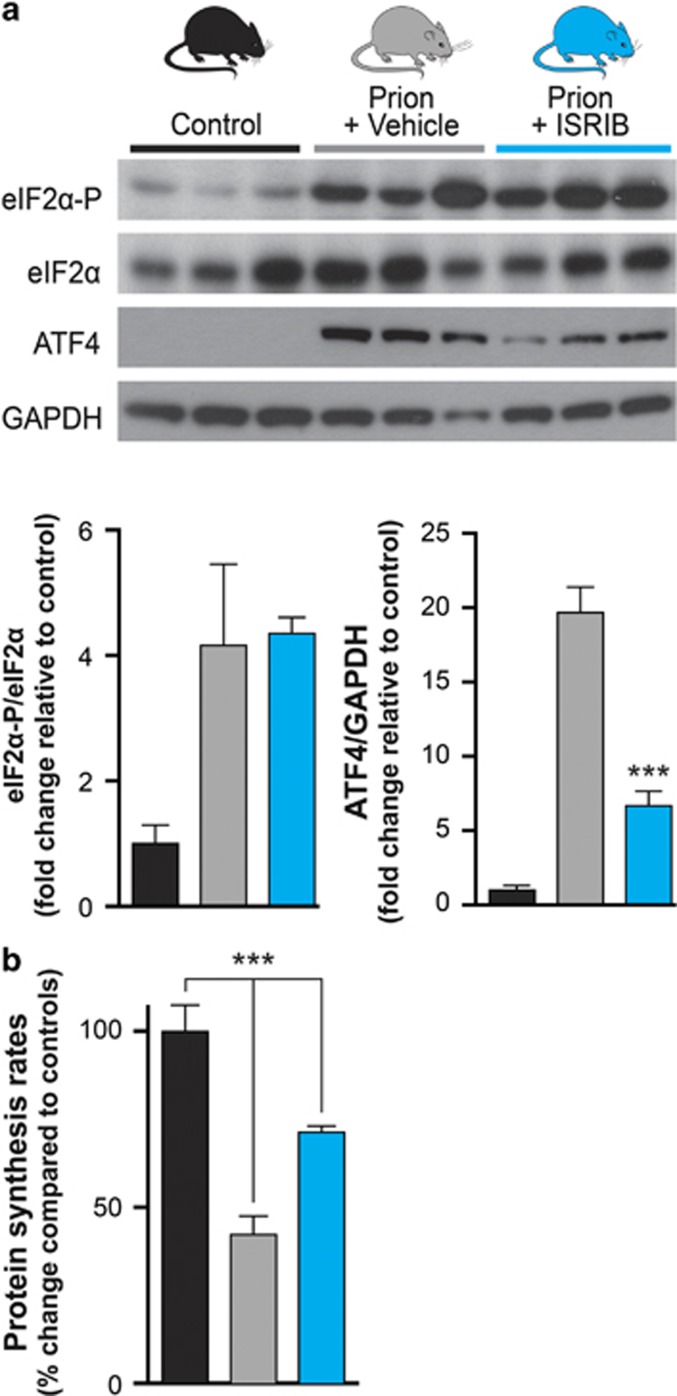
ISRIB restores translation in prion-diseased mice, downstream of eIF2*α* phosphorylation. (**a**) ISRIB treatment (blue bars) lowers ATF4 levels while leaving eIF2*α*-P unchanged in prion-diseased mice when compared with vehicle treated (gray bars) animals, confirming its site of action downstream of eIF2*α*-P. Representative immunoblots of hippocampal lysates and bar chart quantitating relative levels of proteins in three independent samples are shown. (**b**) ISRIB treatment partially restores translation as measured by [^35^S]-methionine incorporation into hippocampal slices, compared with vehicle-treated animals (*n*=3-4 for each group). ***P<0.001, Student's *t-*test, two-tailed. Bar graphs show mean values±S.E.M.

To understand the basis of their diversity, we compared the effects of GSK2606414 and ISRIB on cultured pancreatic cells. To gage the intensity of the ISR, we measured protein synthesis by puromycin incorporation into newly synthesized proteins. As expected, exposure to the ER stress-inducing agent thapsigargin led to marked attenuation in new protein synthesis, which was nearly completely reversed by GSK2606414 (Figure 6a). ISRIB, by contrast, resulted in only partial restoration of protein synthesis in thapsigargin-treated cells (Figure 6a), even at saturating concentrations (Figure 6c), in contrast to GSK2606414 (Figure 6d). These effects matched the *in vivo* profiles of the compounds, where ISRIB treatment led to ~70% recovery of protein synthesis rates (Figure 2b), compared with ~100% reported with GSK2606414 treatment.^10^ Restoration of protein synthesis by both agents was effaced by washing cells free of the compound before thapsigargin application, indicating that both compounds reversibly engage their target *in vivo* (Figure 6a). To further compare the two compounds, ISRIB and GSK2606414 were applied to cells expressing a CHOP::luciferase construct that reflects downstream activation of the ISR-induced CHOP gene^27^ (Figure 1). GSK2606414 treatment completely inhibited tunicamycin-induced CHOP induction, while ISRIB only partially reduced CHOP expression levels measured by assessing relative luminescence induction by the two compounds (Figure 6e), confirming the partial inhibitory effect of ISRIB on UPR/ISR activation.

## Discussion

**Figure 3:**
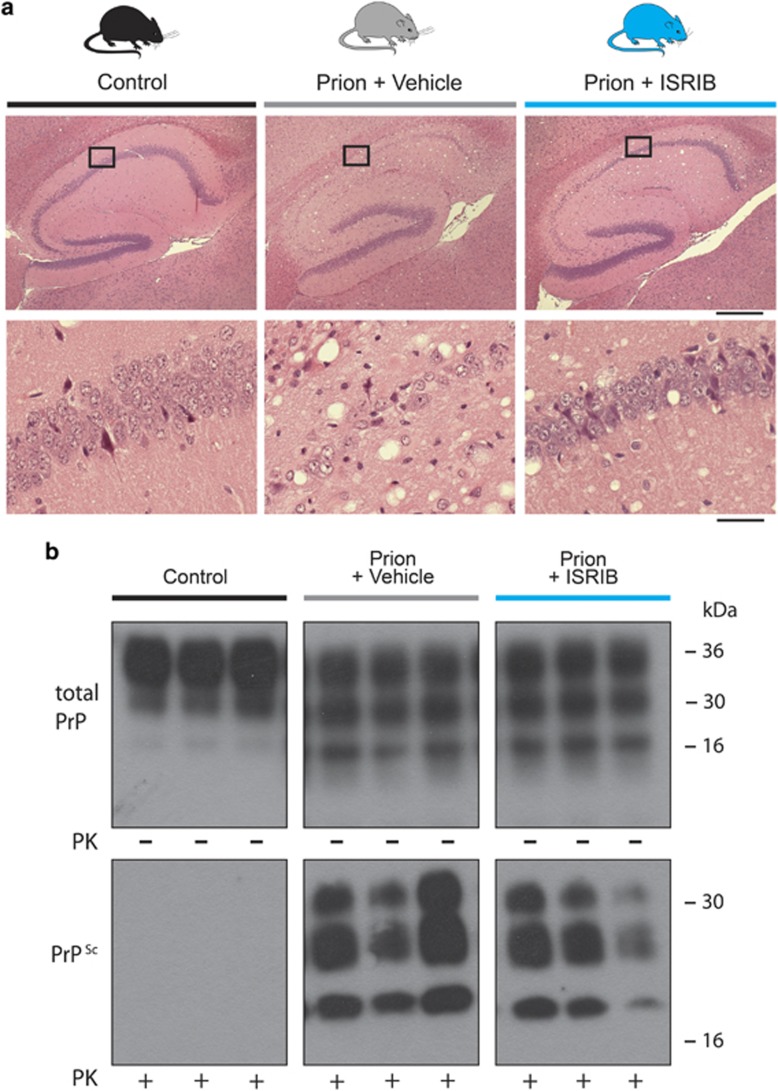
ISRIB confers neuroprotection in prion-diseased mice, via a mechanism independent of prion replication. (**a**) Representative images of hematoxylin and eosin-stained hippocampal sections from uninfected control (left hand panels) and prion-infected mice treated with vehicle (central panels) or ISRIB (right hand panels). Vehicle-treated mice show extensive neuronal loss in the CA1-3 region, with associated spongiosis, while ISRIB treatment prevents neurodegeneration and reduces spongiosis. Scale bar, top row 400 *μ*m, bottom row 50 *μ*m). (**b**) ISRIB treatment does not affect the levels of total PrP and PrP^Sc^. Total PrP and PrP^Sc^ levels, detected with and without proteinase K (PK) digestion, were equivalent in prion-infected mice treated with vehicle or ISRIB. Representative immunoblots of three independent hippocampal lysate samples for total PrP and PrP^Sc^ after PK (50 *μ*g/ml) digestion. Control samples are from mice inoculated with normal brain homogenate

**Figure 4:**
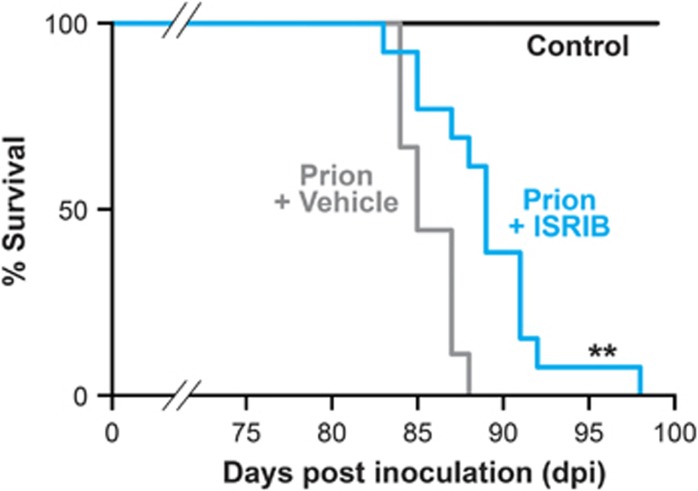
ISRIB treatment significantly extends survival in prion-infected animals compared with vehicle-treated mice. Kaplan–Meier plot, controls *n*=9 (black bar), vehicle *n*=9 (gray bar), ISRIB treated *n*=12 (blue bar). **P<0.005, Mantel–Cox test

**Figure 5:**
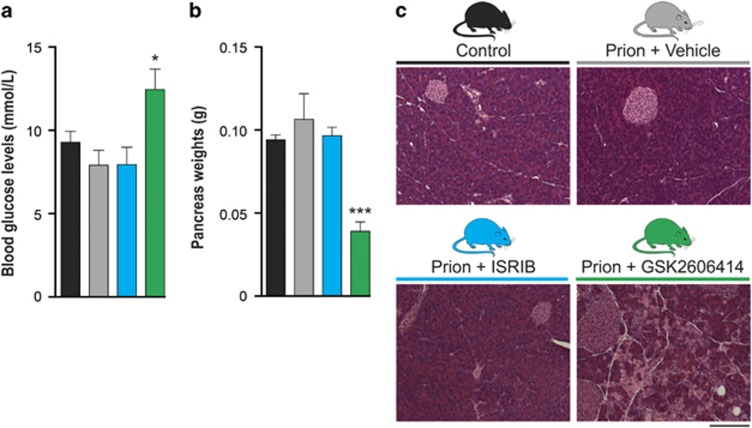
ISRIB is not toxic to the pancreas, unlike GSK2606414. (**a**) GSK2606414 treatment (green bar) mildly raises blood glucose levels compared with control (black bar), vehicle-treated (gray bar) and ISRIB-treated (blue bar) mice (*n*=6–9 for each group). (**b**) GSK2606414 treatment leads to a significant reduction in pancreas weight, while ISRIB treatment has no effect (*n*=3–6 for each group). (**c**) Representative images of hematoxylin and eosin-stained pancreas sections. GSK2606414 treatment leads to extensive destruction of exocrine acinar pancreatic tissue, while ISRIB-treated tissue is histologically normal. Scale bar=200 *μ*m. *P<0.05, ***P<0.001, Student's *t-*test, two-tailed. Bar graphs show mean values±S.E.M.

**Figure 6:**
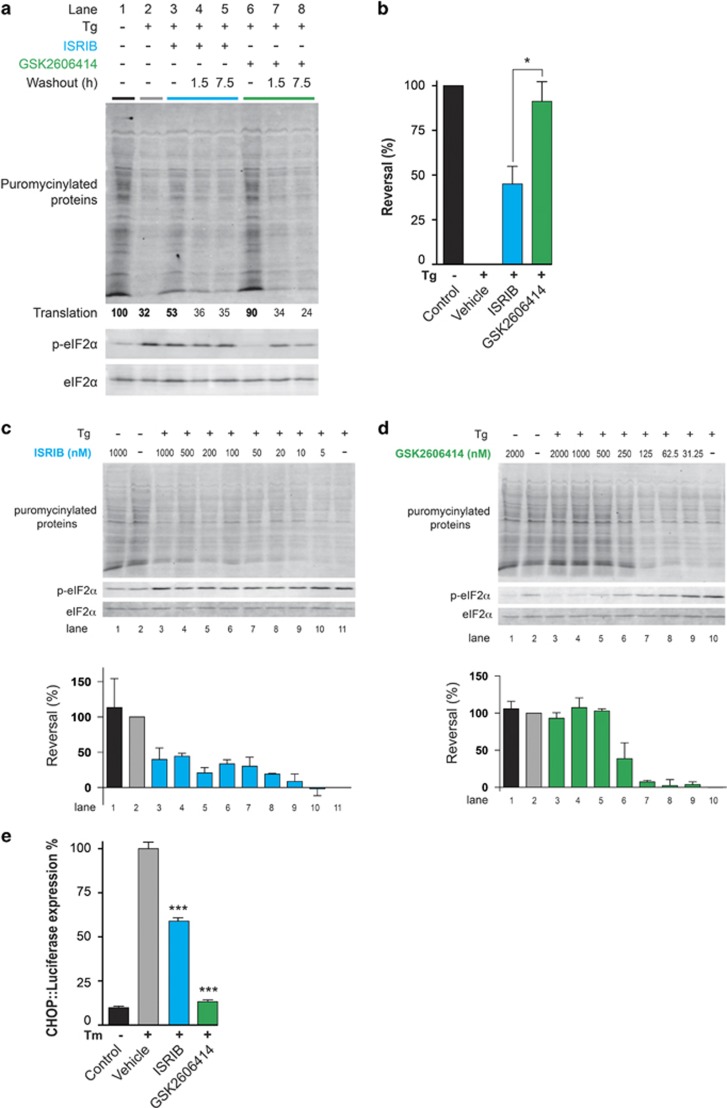
GSK2606414 fully inhibits UPR/ISR-induced translational repression, whereas ISRIB only partially inhibits translational attenuation. (**a**) Immunoblot of puromycinylated (nascent) proteins, phosphorylated eIF2*α* and total eIF2*α* recovered from lysates of AR42j rat pancreatic acinar cells and resolved by SDS–PAGE. Where indicated the cells were exposed to the ER stress-inducing agent thapsigargin (Tg, 0.3 *μ*M) in the presence or absence of ISRIB (100 nM) or the PERK kinase inhibitor GSK2606414 (1 *μ*M) for 30 min before lysis. Puromycin (10 *μ*g/ml) was added during the last 10 min of the incubation. Where indicated (‘Washout') the cells were exposed to the aforementioned concentration of ISRIB or GSK2606414 for 30 min, washed thoroughly with media and incubated further for 1.5 or 7.5 h, before thapsigargin challenge and puromycin labeling. The extent of puromycin labeling (‘Translation') integrated across the surface of each lane is stated below the immunoblot (this value is set arbitrarily to 100 in the reference lane 1, and scaled linearly in the other lanes). (**b**) Reversal of ISR-mediated inhibition of protein synthesis in the experimental conditions described in **a** (Lane 1, 2, 3 and 6) was quantified by integrating the puromycinylated protein signal across the surface of each lane and applying the algebraic operator: *R*^n^=[(*P*^n^−*P*^Tg^)÷(*P*^UT^−*P*^Tg^)] × 100. *R*^n^ is the reversal in lane n. *P*^n^, *P*^UT^ and *P*^Tg^ are the puromycinylated protein signals from the sample of interest (lane n), the untreated sample (lane 1) and the sample exposed to thapsigargin alone (lane 2), respectively. Shown are mean values±S.E.M. (*n*=4, *P<0.05, Student's *t-*test). (**c**) *Blot:* immunoblot of puromycinylated (nascent) proteins, phosphorylated eIF2*α* and total eIF2*α* recovered from lysates of AR42j rat pancreatic acinar cells and resolved by SDS–PAGE. Where indicated the cells were exposed to the ER stress-inducing agent thapsigargin (TG, 0.3 *μ*M) in the presence or absence of the indicated concentrations of ISRIB for 30 min before lysis. Puromycin (10 *μ*g/ml) was added during the last 10 min of the incubation. *Bar chart:* reversal of ISR-mediated inhibition of protein synthesis in the experiment described in ‘**a**' was quantified by integrating the puromycinylated protein signal across the surface of each lane and applying the algebraic operator: *R*^n^=[(*P*^n^−*P*^Tg^)÷(*P*^UT^−*P*^Tg^)] × 100. *R*^n^ is the reversal in lane n. *P*^n^, *P*^UT^ and *P*^Tg^ are the puromycinylated protein signals from the sample of interest (lane n), the untreated sample (lane 2) and the sample exposed to thapsigargin alone (lane 10), respectively. Shown are mean values±variation in an experiment reproduced twice. (**d**) *Blot:* as in ‘**a**' above, but applying the indicated concentrations of GSK2606414. *Bar chart:* as in (**c**) above, but pertaining to GSK2606414. (**e**) Luciferase expression in CHO cells expressing a luciferase reporter construct under control of the CHOP promoter. Tunicamycin treatment leads to robust luciferase expression, which is completely inhibited by GSK2606414 treatment. ISRIB treatment only partially reduces expression (*n*=9 for each group). Shown are mean values±S.E.M, *P<0.05, ***P<0.001 Student's *t-*test, two-tailed

The data show that treatment with ISRIB, as with the PERK kinase inhibitor GSK2606414, is profoundly neuroprotective in prion-diseased mice (Figures 3 and 4). Thus, we have shown that inhibition of UPR/ISR-induced translation repression is beneficial to neurons by treatment with a second small molecule, acting at another point on this pathway. Importantly, however, treatment with ISRIB did not cause pancreatic toxicity, which is seen with GSK2606414, and has been perceived as the major obstacle to therapeutic targeting of the pathway to date (Figure 5). The differential toxicity on the pancreas can be explained by the less extensive inhibition of UPR/ISR activation by ISRIB compared with the effects of PERK inhibition with GSK2606414 (Figure 6), independently of their points of action in the pathway. Thus, ISRIB only restores protein synthesis rates to ~70% of control levels *in vivo* (Figure 2b) and ~50% *in vitro* (Figures 6a and b), even at saturating concentrations of compound (Figure 6c). This is in contrast to GSK2606414, which restores translation rates to ~100% *in vivo*^10^ and 90% *in vitro* (Figures 6a and b). ISRIB essentially acts as a partial inhibitor of eIF2*α*-P-mediated translational repression, although its precise mode and site of action remain unknown.^22^

Together, these observations suggest that the attenuated adverse effect profile of ISRIB compared with GSK2606414 relate to an intrinsic limitation on the extent of ISR inhibition and resultant limited restoration of protein synthesis rates. Thus, the beneficial effects of ISR-reversing drugs in preventing neurodegeneration do not require full suppression of the response.

The problem of weight loss with ISRIB treatment is not explained by pancreatic toxicity, nor by diabetes or other obvious metabolic effect. This may reflect off-target effects of ISRIB, or systemic consequences of persistent prion infection unmasked by the survival of the treated mice. In any case the survival of pancreatic tissue is marked advantage of partial inhibition of the ISR.

The data have important implications for a number of diseases in which the observation of UPR activation occurs, raising the possibility of a generic treatment for multiple disorders. Thus, raised PERK-P and eIF2*α*-P levels are seen in patients with AD, PD and other protein misfolding neurodegenerative disorders,^11, 12, 13, 14, 15^ and there are increasing reports of beneficial UPR/ISR modulation in various neurodegeneration disease models.^28, 29, 30, 31^ Unfortunately, ISRIB is not itself a candidate for such therapies, given its high insolubility, and new compounds are needed. Critically, however, the data support the existence of a therapeutic window of UPR/ISR inhibition that affords neuroprotection without pancreatic toxicity, and mandate the pursuit of drugs that modulate this pathway within this window for the treatment of protein-misfolding neurodegenerative disorders.

## Materials and Methods

### Prion infection of mice

All animal work conformed to UK regulations and institutional guidelines, performed under Home Office guidelines. Tg37^+/−^^23^ mice were inoculated with 1% brain homogenate of Chandler/RML prions aged 3–4 weeks, as described.^24^ Animals were culled when they developed clinical signs of prion disease or lost 20% of body weight from the start of the study. Control mice received 1% normal brain homogenate.

### Compounds

The *trans* isomer of ISRIB was prepared as described.^22^ LC-MS (system: Shimadzu UFLCXR–Applied Biosystems API2000; column: Phenomenex Gemini-NX 3 *μ*m–110 Å-C_18_, 50 x 2 mm, at 40 °C; elution: 0.5 ml/min, 5 to 98% B in A linear gradient over 2 min, then 98% B isocratic for 2 min, where A: 0.1% HCOOH in H_2_O, B: 0.1% HCOOH in MeCN; UV detection at 220 nm), *t*_R_ 2.96 min (*trans*-ISRIB), purity>95% MS (ESI^+^): *m/z* 451.1 [M+H]^+^, C_22_H_26_Cl_2_N_2_O_4_ requires 451.1 MW. NMR in accordance with Sidrauski *et al.*^22^

### Dosing of mice

ISRIB was administered at 0.25 mg/kg once daily by intraperitoneal injection or vehicle alone (45% saline, 50% PEG 400, 5% DMSO) from 7 w.p.i. Treatment with GSK2606414 was by oral gavage twice daily with 50 mg/kg GSK2606414 from 7 w.p.i. as described.^10^

### Immunoblotting

Protein samples were isolated from hippocampi using protein lysis buffer (150 mM NaCl, 1% Triton X-100, 0.5% sodium deoxycholate, 0.1% SDS and 50 mM Tris pH8.0) supplemented with Phos-STOP and protease inhibitors (Roche, Welwyn Garden City, UK). UPR proteins and PrP levels were determined by resolving 20 *μ*g of protein on SDS–PAGE gels, transferred onto nitrocellulose or PVDF membranes and incubated with primary antibodies for total PrP and PrP^Sc^ (ICSM35 1 : 10,000; D-GEN), eIF2*α*-P, eIF2*α* (1 : 1000; Cell Signaling, Leiden, Netherlands), ATF4 (CREB-2, 1 : 1000; Santa Cruz, Santa Cruz, CA, USA). Horseradish peroxidase-conjugated secondary antibodies (1 : 5000; Dako, Glostrup, Denmark) were applied and protein visualized using enhanced chemiluminescence (GE Healthcare, Little Chalfont, UK) and quantitated using ImageJ. Antibodies against GAPDH (1 : 5000; Santa Cruz) were used to determine loading.

### Protein synthesis rates

Protein synthesis rates were calculated by measuring [^35^S]-methionine incorporation into proteins in acute hippocampal slices, as described.^8, 10^ In brief, hippocampal slices were prepared with a tissue chopper (McIlwain) and dissected in an oxygenated cold (2° to 5 °C) sucrose artificial cerebrospinal fluid (ACSF) containing 26 mM NaHCO_3_, 2.5 mM KCl, 4 mM MgCl_2_, 0.1 mM CaCl_2_ and 250 mM sucrose. Slices were allowed to recover in normal ACSF buffer while being oxygenated at 37 °C for 1 h in 95% O_2_/5% CO_2_, and then incubated with 5.7 mBq of [^35^S]-methionine label for 1 h. Samples were washed and homogenized in 1 × passive lysis buffer (Promega, Fitchburg, WI, USA), and proteins were precipitated with 25% trichloroacetic acid (TCA) (Sigma, Gillingham, UK). TCA lysates were then placed on Whatman filters, washed with 70% industrial methylated spirits and acetone, and then placed into scintillation cocktail buffer. Incorporation of radiolabel was measured by scintillation counting (WinSpectral, Wallac, Coventry, UK).

### Histology

Paraffin-embedded brains and pancreases were sectioned at 5 μm and stained with hematoxylin and eosin (H&E) as described.^8, 10^

### Detection of ISRIB by LC-MS/MS

Blood and brain tissue were collected 8 or 24 h after dosing from mice treated with one doses of 0.25, 2.5 or 5 mg/kg *trans*-ISRIB, or vehicle. Blood plasma (up 0.2 ml, exact volume measured) was diluted with water to 0.2 ml and extracted with 0.4 ml of chloroform/methanol 2 : 1. After vortex mixing (10 min) and centrifugation (10 000 g, 10 min), the lower layer was dried with vacuum centrifugation and reconstituted in 50 *μ*l of methanol. Brain tissue (one complete half, about 0.25 g weighed exactly) was homogenized in 0.5 ml of chloroform/methanol 2 : 1 and further processed exactly as the plasma samples. ISRIB quantitative analysis (using external standards) was performed by LC-MS/MS using a 4000 QTRAP mass spectrometer (Applied Biosystems, Foster City, CA, USA) equipped with a turbo ion source and LC series 10 AD VP (Shimadzu). The mobile phase was a water/acetonitrile gradient modified with 0.1% formic acid using a Agilent Poroshell 120 SB-C18 2.1 × 50 mm^2^ (2.7 *μ*m), which was maintained at 40 °C. LC-MS/MS multiple reaction monitoring used a precursor ion of mass/charge ratio (*m/z*) 452 and a product ion of *m/z* 265 in positive electrospray ionization mode for *trans*-ISRIB. Data analysis was performed with Analyst 1.4.1 in the quantitative mode.

### Cell culture

AR42j cells were cultured in DMEM supplemented with 10% Fetal calf serum (FetalClone II, Thermo Scientific, Loughborough, UK), 2 mM l-glutamine, 1 × Penicillin/Streptomycin and 1 × non-essential amino-acid solution and were maintained at 37 °C with 5% CO_2_. CHO-KI CHOP::luciferase cells were cultured in DMEM/F12(Ham) (Gibco, Paisley, UK) supplemented with 10% Fetal calf serum, 2 mM L-glutamine and 1 × Penicillin/Streptomycin, and were maintained at 37 °C with 5% CO_2_.

### Puromycin labeling and immunoblot analysis

The effects of ER stress on puromycinylated protein levels, total and phosphorylated eIF2*α* in cells were determined as previously described.^32^ In brief, 1 × 10^6^ AR42j cells were plated in 60 mm dishes. Two days later, culture media was changed to fresh media, and cells were treated with vehicle (dimethyl sulfoxide) or thapsigargin in the presence or absence of the indicated concentration of inhibitors for 30 min. For puromycin labeling, 10 *μ*g/ml puromycin was added during the last 10 min before harvest. Cells were lysed with lysis buffer (1% Triton X-100, 50 mM Tris-HCl (pH 7.4), 150 mM NaCl, 1 mM EDTA, 10% Glycerol, 2 mM PMSF, 10 *μ*g/ml aprotinin, 4 *μ*g/ml Pepstatin and 4 *μ*M Leupeptin). After centrifugation at 21 130 × *g* for 10 min, supernatants were mixed with SDS–PAGE sample buffer. To detect puromycinylated protein or eIF2*α*, 40 or 13 *μ*g of total protein, respectively, was subjected to 12% SDS–PAGE and transferred onto PVDF membrane. Immunoblot detection was conducted using primary antibodies for puromycinylated protein (described in Schmidt *et al.*^33^), phospho-eIF2alpha-Ser51 (Epitomics, Burlingame, CA, USA) or total eIF2*α*, and IR800 or IR680 conjugated secondary antisera followed by scanning on a Licor Odyssey scanner (Licor, Cambridge, UK). Scanned images were quantified using imageJ software.

### CHOP::luciferase assay

CHO::KI cells stably transfected with a CHOP::luciferase reporter^27^ were plated at a density of 1 × 10^5^ per well in a six-well plate and left to grow overnight. Cells were treated for 6 h with 5 *μ*g/ml tunicamycin or vehicle only (100% DMSO) and then extracted using the Steady-Glo luciferase assay system (Promega) before being quantified using the Glomax 96 microplate luminometer (Promega). ISRIB and GSK2606414 were incubated with 5 *μ*g/ml tunicamycin for 6 h at 1 or 20 *μ*M, respectively, before assaying as above.

### Statistical analyses

Statistical analyses were performed using Prism V6 software (Graphpad, La Jolla, CA, USA) using Student's *t*-test for data sets with normal distribution and a single intervention. ANOVA testing was performed using one-way analysis with Tukey's *post-hoc* test for multiple comparisons. For Kaplan–Meier analysis, Mantel–Cox analysis was used.

## Figures and Tables

**Table 1:** Plasma concentration of *trans*-ISRIB in mice at 8 and 24 h following single intraperitoneal injection at a range of doses

	**Dose mg/kg**	**Time (h)**	**Plasma mean ng/ml±S.D.**	**Brain mean ng/g±S.D.**	**Mean ratio brain: plasma**
Vehicle		8	NQ	NQ	NQ
*Trans*-ISRIB	0.25	8	345.3±44.7	186.9±46.8	0.54
		24	9.9±1.0	16.0±1.6	1.62
	2.5	8	721.6±60.1	254.6±70.3	0.35
		24	44.1±11.6	39.7±12.6	0.9
	5	8	369.7±114.4	160.3±17.6	0.43
		24	70.9±30.6	64.6±9.2	0.91

Abbreviation: NQ, not quantifiable

Wild-type mice were given single doses of *trans*-ISRIB at 0.25, 2.5 and 5 mg/kg by interperitoneal injection, and the plasma and brain concentrations of *trans*-ISRIB were determined at 8 and 24 h after dosing in each case. The determined mean brain:plasma ratios indicated that *trans*-ISRIB (the active isomer) readily crossed the blood–brain barrier, achieving good penetration into brain tissue at both 8 and 24 h. The lack of correlation between dose and the measured concentrations of *trans*-ISRIB in the plasma and brain suggests a limitation to absorption of the compound, possibly due to the poor aqueous solubility of ISRIB. *Trans*-ISRIB at a concentration of 0.25 mg/kg gave favorable brain penetration and was therefore used for all subsequent experiments. Concentrations shown are all mean±S.D. (*n*=3)

**Table 2:** ISRIB treatment prevents clinical signs of prion disease in infected mice

	**Vehicle-treated mice**	**ISRIB-treated mice**
	*n*=9	*n*=12
*Early indicator signs*		
Rigid tail	9/9	7/12
Hind limb clasping	8/9	7/12
Unsustained hunched posture	4/9	0/12
Mild loss of coordination	9/9	7/12
		
*Confirmatory signs*		
Impairment of righting reflex	5/9	0/12
Dragging of limbs (front/hind)	2/9	0/12
Sustained hunched posture	3/9	0/12
		
*Scrapie incubation*		
Number of animals succumbing to prion disease	9/9	0/12

Prion disease is diagnosed by the presence of two confirmatory clinical signs or two early indicator and one confirmatory clinical signs. Nine out of nine vehicle-treated mice exhibited a mixture of early indicator and confirmatory clinical signs, indicating the development of clinical prion disease. Seven out of 12 ISRIB-treated mice exhibited early indicator clinical signs, but none progressed to confirmatory signs and were not clinical diseased, demonstrating the protective effects of ISRIB treatment on prion disease progression (vehicle treated *n*=9, ISRIB treated *n*=12)

## Abbreviations

AD: Alzheimer's disease
ALS: amyotrophic lateral sclerosis
ATF4: activating transcription factor 4
CHOP: C/EBP homologous protein
eIF2*α*: eukaryotic initiation factor 2 alpha
eIF2*α*-P: phosphorylated eukaryotic initiation factor 2 alpha
ER: endoplasmic reticulum
GADD34: growth arrest and DNA damage protein 34
ISR: integrated stress response
ISRIB: integrated stress response inhibitor
PD: Parkinson's disease
PERK: pancreatic ER kinase
PERK-P: phosphorylated pancreatic ER kinase
PrP: prion protein
PrP^Sc^: protease-resistant prion protein
RML: Rocky Mountain Laboratory
UPR: unfolded protein response
w.p.i.: weeks post inoculation
